# The Biochemical Markers Associated with the Occurrence of Coronary Spasm

**DOI:** 10.1155/2019/4834202

**Published:** 2019-09-17

**Authors:** Liang Li, Yong-Ping Jin, Shu-Dong Xia, Chao Feng

**Affiliations:** Department of Cardiology, The Fourth Affiliated Hospital of Zhejiang University, Yiwu, China

## Abstract

Coronary artery spasm (CAS) is one of the mechanisms of angina pectoris. Unlike the diagnosis of acute myocardial infarction which is based on the elevation of cardiac markers, the diagnosis of CAS is difficult and sometimes requires sophisticated and risky provocative test which is not widely accepted in China. There is no well-established biomarker for the diagnosis or prediction of CAS. However, there are some biomarkers proven to be associated with the occurrence of CAS. For example, inflammatory factors including C-reactive protein and cytokines, lipoprotein (a), and cystatin-C might be precipitating factor for CAS. Rho-kinase as a mediator involved in multiple mechanisms of CAS, serotonin, and endothelin-1 as powerful vasoconstrictors leading to vasospasm were all observed being elevated in patients with CAS. Thioredoxin and nitrotyrosine reflected the oxidative status and could be observed to be elevated after the occurrence of CAS. In some cases doubted to be CAS without the evidence of provocative test, the blood test for the biomarkers mentioned above could be useful for the diagnosis of CAS.

## 1. Background

Coronary artery spasm (CAS) is a transient and reversible vasoconstriction of epicardial coronary artery or coronary microvessel which leads to transient ischemia. CAS is one of the mechanisms of angina pectoris and is not accompanied by the increase of myocardial oxygen demand. Since 1959 when Prinzmetal et al., first described this particular form of angina occurring at rest with ST-segment elevation on electrocardiogram, the pathogenesis of CAS has been studied for decades. Now it is widely believed that the hyper-reactivity of smooth muscle cells (SMC) in coronary arteries to vasoconstrictor agents is the key pathogenesis of CAS. The activity of SMC to vasoconstrictors or the tone of SMC is regulated via complicated mechanisms which involve endothelial function, oxidative stress, eNOS, Rho-kinase activity, and inflammation. Calcium channel blockers (CCB) has been proved to be effective for most CAS. However, unlike the diagnosis of acute myocardial infarction which is based on the elevation of cardiac markers and is accurate and relatively easy to perform, the diagnosis of CAS is not that easy and sometimes need sophisticated and risky provocative approaches. In China, most of the diagnosis of CAS is only based on the history of typical attack of nocturnal or resting angina without provocative tests because of its risk, thus a lot of patients might be misdiagnosed or underdiagnosed. There's no well-established biochemical marker which could be used to identify or predict the occurrence or forthcoming CAS, however, there are some markers which had been proven to indicate a higher possibility of CAS. In this paper, the authors reviewed the markers which had been reported to be associated with the occurrence of CAS and sought to make a conclusion of their association with CAS and reliability of their potential use in the diagnosis of CAS.

## 2. Inflammatory Markers

The role of chronic vascular inflammation in atherosclerosis has been established by many studies [1–7]. The elevation of inflammatory markers has been observed in a series of vascular diseases including coronary artery disease, ischemic stroke, and peripheral artery disease [8–18]. C-reaction protein (CRP) was the most common biomarker used to determine the inflammatory status of vascular disease [19]. Some other markers including cytokines, monocyte count, MPO, sCD40L, and MMPs were all reported to be associated with the inflammatory status of atherosclerosis [20].

Inflammation was also widely believed to be potential stimulant of CAS. Post-mortem and animal studies showed the accumulation of inflammatory cells in coronary vasospastic segments [21, 22]. In a recent study, with 18F-FDG PET-CT, Hiroaki Shimokawa, et al. demonstrated that the inflammation in the adventitia and perivascular adipose tissue was more significant in CAS patients [23]. In clinical studies, the elevation of inflammatory markers was also reported, including CRP, IL-6, sCD40L, monocytes and polymorphonuclear neutrophils [24–27]. CRP and sCD40L were reported to be correlated with the presence of both epicardial and microvascular spasm [27]. The specific role of inflammation in the pathogenesis of CAS still remains to be elucidated. Some studies showed that chronic inflammation suppresses the endothelial function and NO activity [28]. Some cytokines including TNF and IL-1 might enhance the expression and activity of RhoA, and lead to the over-sensitization of Ca2+ through the activation of ROCK pathway [29–31].

However, the specificity of inflammation markers in identification of CAS was weak because inflammation is a common predisposing factor for various vascular diseases. A clinical study showed that the plasma hs-CRP was equally increased in both CAS and CAD groups [32]. Besides, as mentioned above, in the study conducted by Hiroaki Shimokawa the results showed that inflammation in the adventitia and perivascular adipose tissue was significant [23], yet it is not clear whether the systemic inflammation was a precipitating factor for CAS or only the local/adjacent inflammatory stimulant contributes to the occurrence of CAS. Furthermore, the results about the predictive value of some inflammatory markers were not totally consistent among clinical studies. For example, although CRP is reported to be associated with CAS by various studies, a Korean study showed that there was no difference of the levels of CRP between patients with and without CAS, while the peripheral monocyte count is an independent marker for predicting CAS [33]. Anyway, more studies are still required to identify whether some inflammatory marker could be used to discriminate CAS from CAD.

## 3. Lipoprotein (a)

Hyperlipidemia has been well established as one of the major risk factors of atherosclerotic vascular diseases by overwhelming evidences. The level of low-density lipoprotein (LDL) is now a major therapeutic target for CAD and ischemic stroke. Although the mechanism of CAS is quite different from that of exertional angina which is caused by the accumulation of atherosclerotic plaques or unstable plaques, some studies showed that lipoprotein might also play an important role in the pathogenesis of CAS.

In a clinical study conducted by Keiichi Tsuchida, et al, the results showed that serum lipoprotein (a) concentration was significantly higher in those patients who had a higher basal tone at the spastic site of coronary arteries and required a significantly lower ergonovine dose to provoke spasm [34]. Similarly, Masami Nishino reported that patients with CAS had higher level of lipoprotein (a) compared with those without CAS [35]. Lipoprotein (a) might contribute to the occurrence of CAS through regulating the endothelium function. It was reported that elevated concentration of lipoprotein (a) was correlated to the vasoconstrictor response to Ach and could impair receptor-mediated endothelial vasodilation [36, 37]. In addition, lipoprotein (a) might play a role in the genesis of thrombotic coronary occlusion, therefore leads to the occurrence of AMI subsequent to coronary spasm [38]. However, in a recent large-scale clinical study conducted by Ahmed Mashaly, no relationship between the elevated lipoprotein (a) level and the vasospastic response to the intracoronary Ach provocation test was identified [39]. It suggested that the role of lipoprotein (a) in the pathogenesis in CAS is far from reaching a consensus.

## 4. Cystatin C

Cystatin C is a cysteine protease inhibitor produced in all nucleated cells and freely infiltrated by the glomeruli. Cystatin C has been proven to be a reliable marker of glomerular filtration rate, especially in the preclinical stage of kidney dysfunction [40]. Two studies in Japan and South Korea showed that a high level of cystatin C was independently associated with the prevalence of CAS, and suggested that cystatin C might be a potential biomarker of CAS [41, 42].

The association between cystatin C and CAS might reflect the role of mild renal dysfunction in the pathogenesis of CAS. It was demonstrated that renal dysfunction could lead to deactivation of endothelial NO synthetase, thus was associated with endothelial dysfunction which was the fundamental mechanism of CAS [43]. Besides, renal dysfunction could cause vascular inflammation, oxidative stress, and atherosclerosis [44], all of which were the contributors of CAS. A clinical study showed that chronic kidney disease stage was a modulator on the association between hs-CRP and CAS. According to the result of this study, the hs-CRP level and monocyte count were associated with CAS independently only in stage 1 of CKD, when the level of cystatin C was a reliable marker [45].

However, just as mentioned before, renal dysfunction especially advanced renal dysfunction was also associated with atherosclerosis and CAD. Studies were still required to identify whether cystatin C could be used as a unique biomarker of CAS differently from CAD.

## 5. Serotonin

Serotonin (5 H-T) is a well-established vasoactive substance released from aggregating platelets. Since it was discovered in 1930s, a series of studies demonstrated its various roles in vascular disease and neuroscience. Most in vitro studies supported its role as a vasoconstrictor while a few studies suggested in some circumstances, serotonin could be a vasodilator [46]. In normal coronary artery system, serotonin has vasodilating effect; when the endothelium is damaged, infused serotonin could lead to strong vasoconstricting effect or spasm as proved by in vitro study [47, 48]. An early clinical study showed that patients with CAS had higher level of serotonin than controls [49]. Another small-size clinical study showed that the level of serotonin was significantly higher in patients with CAS than that in control even during nonischemic interval [50]. A recent study showed that the level of serotonin was significantly higher in patients with CAS and unobstructed arteries [51]. It suggested that serotonin could be identified as a predictive marker of CAS including microvascular spasm. However, most of the studies about the role of serotonin in the pathogenesis of CAS were conducted decades ago. More clinical studies were still required to detect the level of serotonin in different stages of CAS.

## 6. Endothelin-1

Endothelium is the “gatekeeper” of the vessel health. Physiologically, endothelium responds to humoral, neural, and hemodynamic changes, release vasodilators, and leads to coronary dilation. However, under some pathological condition, vasoconstrictors derived from endothelium overcome and lead to coronary spasm [52]. In 1988, endothelin-1 was identified as the first endothelium-derived contractor by Yanagisawa's group [53]. Since then, endothelin-1 and endothelial dysfunction have been the focus of CAS-associated research. Matsuyama showed that the level of endothelin-1 was increased in the coronary sinus during spasm [54]. Kaski and Hoffmann showed that the concentration of circulating plasma endothelin-1 level was raised in patients with CAS in their respective studies [55, 56]. Toyo-oka found that the level of endothelin-1 was significantly higher in spasm-provoked patients than that in nonprovoked patients, and was transiently decreased to normal after spasm resolved [57]. Kyriakides confirmed that endothelin-1 contributed to the total vasomotor tone mostly mediated by ET-A receptor [58]. In a case reported by Vermeltfoort, the treatment with endothelin receptor antagonist bosentan successfully reduced the frequency and severity of chest pain in a patient with CAS [59]. The experiment on porcine model also demonstrated that administration of ET-A receptor antagonist is effective to prevent CAS [60]. Now endothelin-1 is widely believed to be one of the most potent endogenous vasoconstrictors which could initiate and maintain both epicardial and microvascular spasm.

Based on the results of the studies above, endothelin-1 could be identified as a potential marker for the identification or even prediction of CAS. However, the sensitivity and specificity of endothelin-1 in the identification of CAS might be limited. As a powerful vasoactive factor, endothelin-1 is also associated with atherosclerosis, pulmonary hypertension, hypertension, and renal failure [61]. Besides, it was reported that the plasma level of endothelin-1 was only 1/100 of that within the vascular wall [62]. Therefore the change of endothelin-1 level tested by serum test might not reflect the pathological change of the vessels directly. Third, it was reported that endothelin-1 could augment the vasoconstriction caused by other substances. Even low concentration of endothelin-1 might lead to spasm through its contractile response to serotonin and norepinephrine [63].

## 7. Neuropeptide Y

Neuropeptide Y is a bioactive peptide released from sympathetic nervous system along with norepinephrine after sympathetic stimulation, and acts as a co-transmitter and local neuromodulator of several aspects of cardiac function. Neuropeptide has been studied as a potent vasoactive factor for decades. In 1985, Aizawa's group found that the administration of neuropeptide Y resulted in vasoconstriction of canine coronary artery in a dose-response model [64]. They also identified that calcium-channel blockers could attenuate the vasoconstrictor action of Neuropeptide Y [65]. Preito demonstrated that neuropeptide Y induced the contraction of distal coronary arteries more significantly than that of the proximal epicardial arteries of rats [66]. Later for the first time in clinical trials Clarke showed that the infusion of neuropeptide Y lead to transient typical chest pain and electrocardiographic change in patients without significant coronary stenosis and confirmed the constriction of small vessels rather than epicardial arteries caused by neuropeptide Y by arteriography [67]. Recently, more clinical studies identified that endogenous neuropeptide-Y could cause coronary artery constriction especially coronary microvascular spasm, in both patients without coronary stenosis and patients of ST-elevated myocardial infarction [68–70].

However, by now neuropeptide is mostly studied in the background of high sympathetic drive such as myocardial infarction and hyperventilation test. Few studies compared the basic level of neuropeptide between patients with and without CAS. Although its significant correlation with coronary microvascular spasm has been confirmed, it is still very far away from identifying it as a biomarker to predict or identify the presence of coronary microvascular spasm.

## 8. Rho-kinase

Rho-kinase is one of the downstream targets of GTP-binding protein Rho [71]. Previous studies demonstrated that Rho-kinase played a pivotal role in the regulation of many cellular functions and in the pathogenesis of vascular disease [72]. The impact of Rho-kinase activity in CAS was also reported by several clinical studies. Kikuchi demonstrated that Rho-kinase activity in circulating neutrophils determined by the extent of phosphorylation of myosin-binding submits was significantly higher in CAS group than that in control. Besides, after treatment with CCB, Rho-kinase activity was significantly decreased in CAS group [73]. It suggested that Rho-kinase activity in circulating neutrophils could be a useful biomarker of both diagnosis and disease activity assessment of CAS. Later a study in Taiwan also showed that leukocyte Rho-kinase activity predicted the presence and severity of CAS [74]. Another study proved that the activity of Rho-kinase had a distinct circadian variation with a peak in the morning, strongly associated with coronary basal tone and vasoconstriction [75].

Actually, long before that it has been reported CAS including coronary microvascular spasm could be suppressed by fasudil which is a well-established inhibitor of Rho-kinase [76, 77]. Some animal studies demonstrated that Rho-kinase could inhibit the myosin-binding subunit of myosin phosphate, then enhance the phosphorylation of myosin light chain and lead to vasospasm [78]. In addition, Rho-kinase could also mediate hypoxia-induced down-regulation of endothelial NO synthase, vascular SMC proliferation and migration, the monocyte adhesion and spreading on endothelium [72, 79, 80]. Thus, Rho-kinase may be a crucial mediator of endothelial function, NO synthase, and inflammation, all of which are involved in the pathogenesis of CAS. Based on the animal and clinical studies, Rho-kinase activity might become a promising biomarker of CAS, if the measurement could be more simplified.

## 9. Oxidative Stress Markers

Oxidative stress has been identified as a key factor involved in a series of pathological changes including aging, degeneration, cancer, and vascular disease as well. Vascular oxidative stress and subsequent increase of reactive oxygen species (ROS) were proven to be contributors to vascular dysfunction and atherothrombosis through the impairment of endothelial function [81, 82]. There is growing evidence showing that oxidative stress is associated with the occurrence and severity of CAS, and suggest that some oxidative stress markers might be potential biomarkers of CAS [83, 84].

Oxidized-LDL is the oxidized product of LDL and a well-established marker of oxidative distress. The oxidization of LDL leads to the formation of foam cells, proliferation of SMC, and also activation of inflammation. Besides, oxidized-LDL was proven to be an independent determinant of coronary macrovasomotor and microvasomotor responses which were associated with CAS [85]. In a small clinical study, Kugiyama K1 reported that the oxidized-LDL levels in patients with CAS were significantly higher than those in controls and suggested a significant and positive correlation between oxidized-LDL and the vasoconstrictor response of coronary arteries to Ach and impaired vasodilation of endothelium [84]. Malondialdehyde-Modified-LDL as a form of oxidized-LDL was also reported to be associated with the occurrence of CAS [86]. However, the role of oxidized-LDL in the diagnosis the CAS is still controversial. In a study carried out by Yamaguchi, the results showed that the increase of plasma TG-rich lipoproteins and their remnants were more significant markers of CAS than of CAD, while the increase of plasma oxidized-LDL was equal for both CAS and CAD [32].

Thioredoxin is small protein consisting of a redox-active dithiol/disulfide in the active side which functions as an important oxidoreductase widely involved in the defense against excessive generation of reactive oxygen species (ROS) and also in the regulation of fundamental cell functions [87]. The synthesis of thioredoxin is activated under oxidative stress-related conditions such as infections and ischemia [87], and under some chronic vascular disease such as hypertension and atherosclerosis as well [88, 89]. Some clinical studies demonstrated that the plasma level of thioredoxin was increased among patients with CAS with the decrease of antioxidant Vitamin E [90, 91]. Besides, the level of thioredoxin was associated with the activity of CAS [90]. However, the elevation of thioredoxin might probably be the result of ischemia-reperfusion caused by CAS, but not the cause of CAS. Therefore, the level of thioredoxin might be used to identify the severity of CAS without any predicting value before the occurrence of CAS.

Nitrotyrosine is generated from the reaction between tyrosine residues and peroxynitrite. As an oxidative stress marker, nitrotyrosine could decrease the bioavailability of NO and the production of prostacyclin, therefore lead to the decrease of vasorelaxation [92, 93]. In a clinical study, the results showed that serum level of nitrotyrosine was significantly increased after the onset of CAS induced by acetylcholine provocation, and suggested that nitrotyrosine could be a potential biomarker for the diagnosis of CAS [94]. However, similarly to thioredoxin, the elevation of nitrotyrosine level is probably the result of CAS-induced ischemia, but not the cause of CAS. Therefore, it could just be used as the marker of the activity of CAS, but not a predictor for the occurrence of CAS.

## 10. Conclusion

The exploration of the pathogenesis of CAS has lasted for decades. During the exploration, various biochemical markers were studied with several inflammatory markers, oxidative stress markers, lipoprotein (a), cystatin, serotonin, endothelin-1, neuropeptide Y and Rho-kinase identified to be associated with the occurrence of CAS. However, some studies about CRP and lipoprotein (a) showed negative results. We think that the different study design about the time point of blood test before or after the occurrence of CAS, the limited sample size, the difference of races involved in the studies contributed together to the conflicting results. Although the detail of the pathological changes of CAS is not clearly elucidated yet, the roles of the biochemical markers in the pathological changes before and during CAS could be briefly summarized. Inflammation and oxidative stress might be the main background that contributes to the impaired endothelium function and elevated tone of vessels, with lipoprotein (a) and cystatin C also involved in the process of upstream regulation. Rho-kinase might be the key regulator involved in the pathogenesis of CAS. Serotonin and endothelin-1 function as powerful vasoconstrictors and lead to CAS directly. Besides, although the structures of epicardial coronary artery and microvessels are different, the biochemical markers associated with the spasm of different arteries were mostly similar, such as serotonin, endothelin-1, and Rho-kinase. However, neuropeptide Y as a co-transmitter of norepinephrine is the only biochemical marker proven to be associated with microvascular spasm rather than epicardial coronary spasm. It indicates that the pathogenesis associated with epicardial coronary spasm and microvascular spasm might be different to some extent, with the corresponding effective therapies being different too. Among the various biochemical markers associated with the occurrence of CAS, some of them were identified to be elevated after CAS, such as thioredoxin and nitrotyrosine. However, the elevation of them after CAS was not associated with spasm specifically enough. Yet, the blood test for the biomarkers such as serotonin, endothelin-1, Rho-kinase and neuropeptide Y could be useful for the diagnosis of CAS, especially when provocative test is not acceptable or not available. Anyway, more studies are still required to identify their roles and search for new markers for the diagnosis of CAS.
